# Effects of soybean meal level on growth performance of 11- to 25-kg nursery pigs^1,2^

**DOI:** 10.1093/tas/txaa053

**Published:** 2020-05-05

**Authors:** Henrique S Cemin, Mike D Tokach, Steve S Dritz, Jason C Woodworth, Joel M DeRouchey, Robert D Goodband

**Affiliations:** 1 Department of Animal Sciences and Industry, College of Agriculture, Kansas State University, Manhattan, KS; 2 Department of Diagnostic Medicine/Pathobiology, College of Veterinary Medicine, Kansas State University, Manhattan, KS

**Keywords:** caloric efficiency, growth, protein, soybean meal, swine

## Abstract

Four experiments were conducted to determine the effects of increasing soybean meal (SBM) level in diets with or without 25% distillers dried grains with solubles (DDGS) on growth performance of nursery pigs raised in university or commercial facilities. Treatments were arranged in a 2 × 3 factorial with main effects of SBM (27.5%, 32.5%, or 37.5% of the diet) and DDGS (0% or 25% of the diet). A total of 296, 2,502, 4,118, and 711 pigs with initial body weight (BW) of 10.6, 11.7, 12.5, and 12.3 kg were used in Exp. 1, 2, 3, and 4, respectively. There were 10, 16, 13, and 12 replicates per treatment in Exp. 1, 2, 3, and 4, respectively. After weaning, pigs were fed common diets for approximately 21 d. Then, pens of pigs were assigned to treatments in a randomized complete block design with BW as the blocking factor and experimental diets were fed for 21 d. Pigs were weighed and feed disappearance measured to calculate average daily gain (ADG), average daily feed intake (ADFI), gain-to-feed ratio (G:F), and caloric efficiency (CE). Data were analyzed using the GLIMMIX procedure of SAS with block as a random effect and treatment as a fixed effect. Single degree-of-freedom contrasts were constructed to test the linear and quadratic effects of increasing SBM and their interactions with DDGS. Pigs used in all experiments did not undergo major health challenges during the experimental period and due to the low number of mortality and cull events, statistical analysis was not performed on these variables. The average cull rate was 0.7%, 0.5%, 0.2%, and 0%, and the mortality rate was 0.7%, 0.3%, 0.4%, and 0% in Exp. 1–4, respectively. There were interactions (*P* ≤ 0.039) between SBM and DDGS for G:F and CE in Exp. 2 and for ADG and ADFI in Exp. 3. These were mostly driven by increasing SBM negatively affecting performance in a greater magnitude when diets contained DDGS compared to diets without DDGS. The main effects of DDGS and SBM were more consistently observed across experiments. Pigs fed diets with 25% DDGS had decreased (*P* ≤ 0.001) ADG and ADFI in all experiments, as well as poorer (*P* ≤ 0.028) G:F and CE except for Exp. 3. Feeding increasing amounts of SBM generally did not result in any major impact in ADG but consistently improved (linear, *P* ≤ 0.078) G:F and CE across experiments.

## INTRODUCTION

Soybean meal (SBM) is the primary plant-protein source for swine diets in the United States (Stein et al., 2013; Pettigrew et al., 2017). The amino acid (AA) profile of SBM is highly digestible and complements major dietary cereal grain AA profiles, such as those of corn and wheat (NRC, 2012). Moreover, the processing techniques to remove SBM antinutritional factors are well described and consistent. Additionally, research suggests health benefits when feeding high SBM levels. Trials with nursery (Rocha et al., 2013; Rochell et al., 2015) and finishing pigs (Johnston et al., 2010) infected with porcine reproductive and respiratory syndrome (PRRS) suggest that health-challenged pig growth performance is improved by feeding high SBM levels. Although the mechanisms are not fully understood, it is suggested that SBM bioactive compounds, namely isoflavones and saponins, may be involved in this response (Smith and Dilger, 2018).

Distillers dried grains with solubles (DDGS) is a coproduct of the ethanol industry widely used in swine diets. It is generally accepted that 30% DDGS can be included in late nursery diets without significantly compromising growth performance (Stein and Shurson, 2009), although factors such as fat and fiber content and mycotoxin levels must be considered. Diets today are frequently formulated with higher amounts of DDGS amounts and increasing feed-grade AA replacing intact protein sources, such as SBM, which typically reduces diet costs. However, given the potential benefits of SBM, a minimum amount may be desirable. We hypothesize that SBM may be especially beneficial for pigs raised under the rigors of commercial conditions. Therefore, the objective of the current study was to determine the effects of increasing SBM in diets with or without DDGS on growth performance of 11- to 25-kg nursery pigs across different environmental conditions.

## MATERIAL AND METHODS

The Kansas State University Institutional Animal Care and Use Committee approved the protocol used in these experiments.

### Ingredients and Chemical Analysis

Samples of corn, SBM, and DDGS were obtained from each location and submitted to the Agricultural Experimental Station Chemical Laboratories (University of Missouri-Columbia, Columbia, MO) for total AA content analysis (method 982.30; AOAC International, 2006) prior to diet formulation (Table 1). The total AA values for corn and SBM were multiplied by NRC (2012) standardized ileal digestible (SID) coefficients and used in diet formulation. Corn, SBM, and DDGS were also analyzed (Ward Laboratories, Inc., Kearney, NE) for dry matter (method 935.29; AOAC International, 1990), crude protein (method 990.03; AOAC International, 1990), neutral detergent fiber (Ankom, 1998), and ether extract (Ankom, 2004). Samples of DDGS from each location were analyzed (North Dakota State University Veterinary Diagnostic Laboratory, Fargo, ND) for mycotoxin concentrations through extraction in acetonitrile and water followed by liquid chromatography with tandem mass spectrometry (LC/MS/MS) detection (Table 2).

**Table 1.** Proximate and total amino acid analysis of soybean meal, DDGS, and corn (as-fed basis)^*a*^

	Soybean meal				DDGS				Corn			
Item, %	Exp. 1	Exp. 2	Exp. 3	Exp. 4	Exp. 1	Exp. 2	Exp. 3	Exp. 4	Exp. 1^*b*^	Exp. 2	Exp. 3	Exp. 4
Dry matter	89.7	89.5	88.84	88.4	90.0	90.8	89.1	89.5	88.3	87.8	89.2	85.9
Crude protein	47.5	47.5	48.5	47.6	31.2	28.7	27.5	27.2	8.2	6.3	7.3	7.0
Neutral detergent fiber	8.1	8.0	6.7	9.7	25.5	27.9	30.6	30.5	9.1	7.0	5.2	6.8
Ether extract	1.4	1.5	1.7	1.6	6.6	8.8	6.9	7.1	3.5	3.6	3.7	2.8
Calcium	0.61	0.54	0.54	0.62	0.10	0.08	0.06	0.06	0.02	0.07	0.07	0.04
Phosphorus	0.71	0.69	0.63	0.60	1.01	0.88	0.91	0.84	0.26	0.23	0.23	0.20
AA												
Alanine	2.02	2.07	2.08	2.01	2.75	1.86	1.85	1.85	0.60	0.45	0.52	0.48
Arginine	3.40	3.46	3.39	3.34	1.58	1.27	1.25	1.22	0.37	0.30	0.34	0.28
Aspartic acid	5.24	5.43	5.39	5.25	2.46	1.79	1.79	1.81	0.54	0.44	0.48	0.43
Cysteine	0.73	0.73	0.69	0.69	0.80	0.60	0.60	0.65	0.19	0.16	0.18	0.16
Glutamic acid	8.34	8.69	8.64	8.29	6.05	3.64	4.18	4.20	1.48	1.11	1.27	1.15
Glycine	1.97	2.00	2.04	1.96	1.49	1.11	1.13	1.16	0.31	0.26	0.29	0.28
Histidine	1.23	1.26	1.22	1.22	1.06	0.78	0.79	0.79	0.24	0.19	0.21	0.19
Isoleucine	2.28	2.30	2.31	2.26	1.60	1.09	1.04	1.07	0.28	0.24	0.26	0.24
Leucine	3.59	3.70	3.66	3.58	4.90	3.19	3.02	3.10	0.96	0.71	0.83	0.75
Lysine	3.05	3.14	3.03	3.01	1.22	1.08	1.04	1.04	0.25	0.25	0.26	0.24
Methionine	0.67	0.67	0.65	0.63	0.80	0.50	0.53	0.53	0.18	0.13	0.14	0.14
Phenylalanine	2.44	2.52	2.46	2.39	2.21	1.69	1.33	1.27	0.39	0.31	0.35	0.31
Proline	2.36	2.48	2.42	2.26	3.04	2.07	2.20	2.25	0.71	0.56	0.59	0.56
Serine	2.03	2.19	2.12	1.94	1.66	1.26	1.14	1.11	0.38	0.29	0.33	0.28
Threonine	1.80	1.88	1.83	1.79	1.46	1.10	1.02	1.05	0.28	0.23	0.27	0.24
Tryptophan	0.72	0.69	0.62	0.67	0.38	0.22	0.18	0.21	0.06	0.06	0.05	0.06
Tyrosine	1.74	1.77	1.51	1.61	1.51	1.03	1.06	0.90	0.26	0.18	0.18	0.13
Valine	2.32	2.38	2.40	2.34	2.08	1.45	1.38	1.37	0.38	0.31	0.34	0.32

^*a*^A representative sample of each ingredient was obtained, homogenized, and submitted to the Agricultural Experimental Station Chemical Laboratories (University of Missouri-Columbia, Columbia, MO) for AA analysis and Ward Laboratories (Kearney, NE) for proximate analysis prior to diet formulation.

^*b*^ NRC (2012) AA values were used for corn in Exp. 1.

**Table 2.** Mycotoxin analysis of distillers dried grains with solubles^*a*^

Mycotoxins	Practical quantitation limit, ppb	Exp. 1	Exp. 2	Exp. 3	Exp. 4
Aflatoxin B1	20	<20	<20	<20	<20
Aflatoxin B2	20	<20	<20	<20	<20
Aflatoxin G1	20	<20	<20	<20	<20
Aflatoxin G2	20	<20	<20	<20	<20
Deoxynivalenol	200	1,047	825	4,093	4,231
Fumonisin B1	200	5,031	214	961	895
Fumonisin B2	200	1,316	<200	244	244
HT-2 toxin	200	<200	<200	<200	<200
Ochratoxin A	20	<20	<20	<20	<20
T-2 toxin	20	<20	<20	<20	<20
Sterigmatocystin	20	<20	<20	<20	<20
Zearalenone	100	111	<100	328	274

^*a*^A representative sample of each source was collected, homogenized, and submitted to North Dakota State University Veterinary Diagnostic Laboratory (Fargo, ND).

Representative diet samples were obtained from each treatment within experiment and stored at −20 °C until analysis. Samples were analyzed (Ward Laboratories, Inc., Kearney, NE) for dry matter, crude protein, calcium (method 985.01; AOAC International, 1990), phosphorus (method 985.01; AOAC International, 1990), neutral detergent fiber, and ether extract.

### Animals and Diets

A total of four experiments were conducted, one in a university facility and three in commercial research facilities. In all experiments, pigs were weaned at approximately 21 d of age, placed in pens based on initial body weight (BW), and fed common diets for approximately 21 d. On day 21, which was considered day 0 of the trials, pens of pigs were allotted to one of six dietary treatments in a randomized complete block design with BW as the blocking factor. Treatments were arranged in a 2 × 3 factorial with main effects of SBM (27.5%, 32.5%, or 37.5% of the diet) and DDGS (0% or 25% of the diet). The increasing levels of SBM were obtained by changing the amount of feed-grade AA and corn. Diets (Tables 3–6) were formulated to contain the same net energy (NE). The NE value for DDGS was estimated as a function of the oil content based on Graham et al. (2014) equation. The NE of SBM used in diet formulation was 88% of corn NE (as-fed basis) or 2,351 kcal/kg NE. Diets were provided ad libitum in mash form. There were 10, 16, 13, and 12 replicates per treatment in Exp. 1, 2, 3, and 4, respectively.

**Table 3.** Diet composition of Exp. 1 (as-fed basis)

	0% DDGS			25% DDGS		
Item	27.5% SBM	32.5% SBM	37.5% SBM	27.5% SBM	32.5% SBM	37.5% SBM
Ingredient, %						
Corn	66.67	61.76	56.86	40.66	35.69	30.71
Soybean meal	27.52	32.51	37.48	27.50	32.52	37.50
DDGS	—	—	—	25.00	25.00	25.00
Choice white grease	1.60	2.00	2.40	3.80	4.15	4.50
Calcium carbonate	0.80	0.78	0.75	1.18	1.15	1.13
Monocalcium phosphate, 21.5% P	1.03	0.95	0.90	0.30	0.23	0.15
Sodium chloride	0.68	0.68	0.68	0.50	0.50	0.50
L-Lysine HCl	0.545	0.385	0.225	0.400	0.240	0.080
DL-Methionine	0.225	0.180	0.130	0.070	0.025	—
L-Threonine	0.280	0.215	0.150	0.140	0.075	0.010
L-Tryptophan	0.065	0.035	0.005	0.030	—	—
L-Valine	0.175	0.090	—	—	—	—
Vitamin premix^*a*^	0.250	0.250	0.250	0.250	0.250	0.250
Trace-mineral premix^*b*^	0.150	0.150	0.150	0.150	0.150	0.150
Phytase^*c*^	0.025	0.025	0.025	0.025	0.025	0.025
Total	100.0	100.0	100.0	100.0	100.0	100.0
Calculated analysis						
SID amino acids, %						
Lysine	1.30	1.30	1.30	1.30	1.30	1.30
Isoleucine:lysine	55	62	69	67	74	81
Leucine:lysine	110	119	128	149	158	167
Methionine:lysine	38	36	34	32	30	30
Methionine and cystine:lysine	58	58	58	58	58	60
Threonine:lysine	65	65	65	65	65	65
Tryptophan:lysine	21.3	21.3	21.4	21.2	21.2	23.6
Valine:lysine	72	72	72	75	81	88
Histidine:lysine	34	37	41	42	45	49
NE, kcal/kg	2,590	2,590	2,590	2,590	2,590	2,590
Crude protein, %	19.5	21.2	22.8	24.7	26.4	28.2
Neutral detergent fiber, %	8.3	8.3	8.2	12.3	12.3	12.2
Calcium, %	0.74	0.74	0.75	0.78	0.78	0.79
STTD P, %	0.45	0.45	0.45	0.45	0.45	0.45
Analyzed values, %						
Dry matter	90.2	90.0	90.3	90.8	90.9	90.5
Crude protein	19.9	21.7	21.9	23.9	25.9	28.4
Neutral detergent fiber	5.2	5.5	5.5	13.4	12.8	13.5
Ether extract	4.3	4.7	5.0	7.8	8.0	7.9
Calcium	0.79	0.73	0.87	1.02	0.90	1.01
Phosphorus	0.53	0.56	0.60	0.61	0.60	0.62

STTD P, standardized total tract digestible phosphorus.

^*a*^Provided per kilogram of premix: 1,654,468 IU vitamin A; 661,387 IU vitamin D; 17,637 IU vitamin E; 1,323 mg vitamin K; 13.2 mg vitamin B12; 19,842 mg niacin; 11,023 mg pantothenic acid; 3,307 mg riboflavin.

^*b*^Provided per kilogram of premix: 73 g Zn from zinc sulfate; 73 g Fe from ferrous sulfate; 22 g Mn from manganese oxide; 11 g Cu from copper sulfate; 0.2 g I from calcium iodate; 0.2 g Se from sodium selenite.

^*c*^Ronozyme HiPhos 2700 (DSM Nutritional Products, Inc., Parsipanny, NJ).

**Table 4.** Diet composition of Exp. 2 (as-fed basis)

	0% DDGS			25%DDGS		
Item	27.5% SBM	32.5% SBM	37.5% SBM	27.5% SBM	32.5% SBM	37.5% SBM
Ingredient, %						
Corn	66.81	61.91	56.91	42.07	37.08	32.05
Soybean meal	27.49	32.48	37.49	27.50	32.50	37.50
DDGS	—	—	—	25.00	25.00	25.00
Beef tallow	1.60	2.00	2.45	2.45	2.85	3.25
Calcium carbonate	0.80	0.78	0.75	1.18	1.15	1.13
Monocalcium phosphate, 21.5% P	1.03	0.95	0.90	0.30	0.23	0.15
Sodium chloride	0.68	0.68	0.68	0.50	0.50	0.50
L-Lysine HCl	0.513	0.352	0.190	0.365	0.204	0.043
DL-Methionine	0.260	0.210	0.165	0.115	0.065	0.020
L-Threonine	0.285	0.215	0.145	0.135	0.065	—
L-Tryptophan	0.073	0.045	0.015	0.045	0.018	—
L-Valine	0.205	0.125	0.025	—	—	—
Vitamin trace-mineral premix^*a*^	0.150	0.150	0.150	0.150	0.150	0.150
Phytase^*b*^	0.015	0.015	0.015	0.015	0.015	0.015
Total	100.0	100.0	100.0	100.0	100.0	100.0
Calculated analysis						
SID amino acids, %						
Lysine	1.30	1.30	1.30	1.30	1.30	1.30
Isoleucine:lysine	54	61	68	66	73	79
Leucine:lysine	101	111	121	141	150	160
Methionine:lysine	38	36	35	33	31	29
Methionine and cystine:lysine	58	58	58	58	58	58
Threonine:lysine	65	65	65	65	65	65
Tryptophan:lysine	21.3	21.4	21.3	21.3	21.4	22.2
Valine:lysine	73	73	73	73	80	87
Histidine:lysine	32	36	40	41	44	48
NE, kcal/kg	2,590	2,590	2,590	2,590	2,590	2,590
Crude protein, %	18.2	20.0	21.7	23.3	25.1	26.9
Neutral detergent fiber, %	8.8	8.6	8.4	13.2	12.9	12.7
Calcium, %	0.71	0.72	0.74	0.74	0.75	0.76
STTD P, %	0.45	0.45	0.45	0.45	0.45	0.45
Analyzed values, %						
Dry matter	88.6	89.0	89.0	90.6	90.4	91.1
Crude protein	19.0	19.7	21.8	22.0	25.2	28.2
Neutral detergent fiber	7.4	6.4	5.7	10.9	12.3	13.2
Ether extract	4.2	3.9	4.3	5.7	6.5	6.6
Calcium	0.62	0.61	0.67	0.72	0.67	0.56
Phosphorus	0.55	0.52	0.56	0.53	0.59	0.59

STTD P, standardized total tract digestible phosphorus.

^*a*^Provided per kilogram of premix: 5,344,543 IU vitamin A; 1,336,137 IU vitamin D; 100,211 IU vitamin E; 1,671 mg vitamin K; 21.4 mg vitamin B12; 29,061 mg niacin; 15,366 mg pantothenic acid; 4,008 mg riboflavin; 66.8 mg biotin; 668 mg folic acid; 1202 mg vitamin B6; 73 g Zn from zinc sulfate; 67 g Fe from ferrous sulfate; 27 g Mn from manganese oxide; 10 g Cu from copper sulfate; 0.5 g I from calcium iodate; 0.2 g Se from sodium selenite.

^*b*^Optiphos 2000 (Huvepharma, Inc., Peachtree City, GA).

**Table 5.** Diet composition of Exp. 3 (as-fed basis)

	0% DDGS			25% DDGS		
Item	27.5% SBM	32.5% SBM	37.5% SBM	27.5% SBM	32.5% SBM	37.5% SBM
Ingredient, %						
Corn	66.34	61.51	56.56	40.64	35.66	30.68
Soybean meal	27.50	32.50	37.50	27.50	32.50	37.50
DDGS	—	—	—	25.00	25.00	25.00
Corn oil	1.60	1.95	2.35	3.45	3.80	4.15
Calcium carbonate	0.85	0.83	0.80	1.23	1.20	1.20
Monocalcium phosphate, 21.5% P	1.15	1.05	1.00	0.45	0.40	0.33
Sodium chloride	0.50	0.50	0.50	0.33	0.33	0.33
L-Lysine HCl	0.547	0.387	0.228	0.408	0.249	0.090
DL-Methionine	0.255	0.210	0.165	0.090	0.045	0.000
L-Threonine	0.280	0.215	0.150	0.150	0.080	0.015
L-Tryptophan	0.095	0.065	0.040	0.065	0.040	0.010
L-Valine	0.185	0.090	0.000	0.000	0.000	0.000
Vitamin trace-mineral premix^*a*^	0.175	0.175	0.175	0.175	0.175	0.175
Phytase^*b*^	0.025	0.025	0.025	0.025	0.025	0.025
Sodium metabisulfite	0.500	0.500	0.500	0.500	0.500	0.500
Total	100.0	100.0	100.0	100.0	100.0	100.0
Calculated analysis						
SID amino acids, %						
Lysine	1.30	1.30	1.30	1.30	1.30	1.30
Isoleucine:lysine	54	61	69	65	72	80
Leucine:lysine	105	115	124	139	149	159
Methionine:lysine	38	36	34	31	30	28
Methionine and cystine:lysine	57	57	57	56	56	57
Threonine:lysine	65	65	65	65	65	65
Tryptophan:lysine	21.2	20.9	21.1	20.6	20.7	20.4
Valine:lysine	72	72	72	73	80	87
Histidine:lysine	32	36	39	41	44	48
NE, kcal/kg	2,590	2,590	2,590	2,590	2,590	2,590
Crude protein, %	19.2	21.0	22.7	23.8	25.6	27.4
Neutral detergent fiber, %	5.3	5.4	5.4	11.6	11.7	11.7
Calcium, %	0.72	0.72	0.72	0.74	0.75	0.76
STTD P, %	0.45	0.45	0.45	0.45	0.45	0.45
Analyzed values, %						
Dry matter	87.8	88.0	88.3	88.4	88.2	88.4
Crude protein	19.2	19.2	21.3	20.8	24.2	26.7
Neutral detergent fiber	7.1	6.8	6.8	11.3	13.1	13.2
Ether extract	4.4	4.7	4.8	7.3	7.4	7.3
Calcium	0.74	0.78	0.79	0.97	0.83	0.97
Phosphorus	0.52	0.56	0.53	0.57	0.58	0.56

STTD P, standardized total tract digestible phosphorus.

^*a*^Provided per kilogram of premix: 1,653,468 IU vitamin A; 551,156 IU vitamin D; 17,637 IU vitamin E; 1,323 mg vitamin K; 13.2 mg vitamin B12; 22,046 mg niacin; 11,023 mg pantothenic acid; 3,086 mg riboflavin; 88 g Zn from zinc sulfate; 77 g Fe from ferrous sulfate; 6.6 g Mn from manganese oxide; 9.9 g Cu from copper sulfate; 0.2 g I from calcium iodate; 0.2 g Se from sodium selenite.

^*b*^Quantum Blue 2500 (AB Vista, Marlborough, UK).

**Table 6.** Diet composition of Exp. 4 (as-fed basis)

	0% DDGS			25% DDGS		
Item	27.5% SBM	32.5% SBM	37.5% SBM	27.5% SBM	32.5% SBM	37.5% SBM
Ingredient, %						
Corn	66.15	61.17	56.33	40.26	35.23	30.21
Soybean meal	27.51	32.52	37.51	27.52	32.52	37.52
DDGS	—	—	—	25.00	25.00	25.00
Corn oil	1.80	2.25	2.60	3.80	4.20	4.60
Calcium carbonate	0.75	0.73	0.70	1.10	1.08	1.05
Monocalcium phosphate, 21.5% P	1.20	1.15	1.05	0.55	0.50	0.45
Sodium chloride	0.50	0.50	0.50	0.35	0.35	0.35
L-Lysine HCl	0.565	0.406	0.247	0.422	0.264	0.105
DL-Methionine	0.280	0.235	0.190	0.110	0.070	0.025
L-Threonine	0.305	0.235	0.165	0.165	0.100	0.030
L-Tryptophan	0.085	0.055	0.025	0.060	0.030	0.000
L-Valine	0.185	0.095	0.015	0.000	0.000	0.000
Vitamin premix^*a*^	0.050	0.050	0.050	0.050	0.050	0.050
Trace-mineral premix^*b*^	0.090	0.090	0.090	0.090	0.090	0.090
Phytase^*c*^	0.025	0.025	0.025	0.025	0.025	0.025
Sodium metabisulfite	0.500	0.500	0.500	0.500	0.500	0.500
Total	100.0	100.0	100.0	100.0	100.0	100.0
Calculated analysis						
SID amino acids, %						
Lysine	1.30	1.30	1.30	1.30	1.30	1.30
Isoleucine:lysine	53	60	67	64	71	78
Leucine:lysine	100	110	119	137	147	156
Methionine:lysine	39	38	36	32	31	29
Methionine and cystine:lysine	58	58	58	58	58	58
Threonine:lysine	65	65	64	65	65	65
Tryptophan:lysine	21.8	21.7	21.6	21.8	21.7	21.6
Valine:lysine	71	71	71	71	78	85
Histidine:lysine	31	35	39	40	44	47
NE, kcal/kg	2,590	2,590	2,590	2,590	2,590	2,590
Crude protein, %	18.8	20.5	22.3	23.3	25.1	26.9
Neutral detergent fiber, %	7.9	8.0	8.1	13.5	13.6	13.7
Calcium, %	0.69	0.70	0.70	0.72	0.73	0.74
STTD P, %	0.45	0.45	0.45	0.45	0.45	0.45
Analyzed values, %						
Dry matter	87.8	88.0	88.1	88.5	88.7	88.8
Crude protein	17.2	18.8	22.2	23.4	25.3	25.8
Neutral detergent fiber	6.5	6.1	5.9	12.6	12.9	12.4
Ether extract	4.2	4.1	4.7	6.8	7.3	7.6
Calcium	0.53	0.61	0.72	0.67	0.89	0.79
Phosphorus	0.49	0.52	0.61	0.62	0.61	0.62

STTD P, standardized total tract digestible phosphorus.

^*a*^Provided per kilogram of premix: 28,660,117 IU vitamin A; 4,409,249 IU vitamin D; 105,822 IU vitamin E; 8,009 mg vitamin K; 79.4 mg vitamin B12; 308,647 mg niacin; 66,139 mg pantothenic acid; 15,432 mg riboflavin.

^*b*^Provided per kilogram of premix: 112 g Zn from zinc sulfate; 104 g Fe from ferrous sulfate; 30 g Mn from manganese sulfate; 16 g Cu from copper sulfate; 0.16 g I from ethylenediamine dihydriodide; 0.2 g Se from sodium selenite.

^*c*^Quantum Blue 2500 (AB Vista, Marlborough, UK).

Experiment 1 was conducted at the Kansas State University Swine Teaching and Research Center (Manhattan, KS). A total of 296 pigs (DNA 241 × 600, Columbus, NE; initially 10.6 kg) were placed in pens of 4 or 5 mixed gender pigs each and used in a 24-d trial. Pens (1.52 × 1.52 m) had metal slatted floors and were equipped with a four-hole stainless steel dry feeder and a nipple waterer. Experiment 2 was conducted at New Horizon Farms Nursery Research (Pipestone, MN). In Exp. 2, 2,502 pigs (PIC 337 × 1050, Hendersonville, TN; initially 11.7 kg) were placed in pens with 24–27 mixed gender pigs each and used in a 21-d trial. Each pen (3.7 × 2.3 m) had plastic floors and was equipped with a six-hole stainless steel dry feeder and a pan waterer. Experiment 3 was conducted at Hord Family Farms nursery research facility (Bucyrus, OH). A total of 4,118 pigs (PIC 337 × 1050, Hendersonville, TN; initially 12.5 kg) were used in a 21-d trial. Two pens sharing a fence line feeder were considered the experimental unit and had 48–54 mixed gender pigs each. Pens (2.3 × 2.7 m) had plastic slatted floor and were equipped with a double-sided five-hole stainless steel feeder and a cup waterer. Experiment 4 was conducted at the Cooperative Research Farm’s Swine Research Nursery (Kalmbach Feeds, Inc., Sycamore, OH). A total of 711 pigs (PIC 380 ×1050, Hendersonville, TN; initially 12.3 kg) were placed in pens with 9 or 10 mixed gender pigs and used in a 21-d trial. Each pen (1.52 × 1.83 m) had slatted metal floors and was equipped with a four-hole stainless steel dry feeder and a nipple-cup waterer.

In all experiments, pens of pigs were weighed and feed disappearance was measured weekly to calculate average daily gain (ADG), average daily feed intake (ADFI), and gain-to-feed ratio (G:F). Mortality and culls were recorded daily. Caloric efficiency (CE) was calculated by multiplying ADFI by kilocalories of NE per kilogram of diet and dividing by ADG.

### Statistical Analysis

Data were analyzed as a randomized complete block design in a 2 × 3 factorial treatment arrangement. There was significant treatment × experiment interaction; thus, each experiment was analyzed separately. Single degree-of-freedom contrasts were constructed to test the linear and quadratic effects of increasing SBM and their interactions with DDGS. Block was included as a random effect and treatment as a fixed effect. Pen was considered the experimental unit in all experiments except in Exp. 3 where two pens shared a feeder; the feeder was considered the experimental unit. Data were analyzed using the GLIMMIX procedure of SAS 9.4 (SAS Institute Inc., Cary, NC). Results were considered significant at *P* ≤ 0.05 and a tendency at 0.05 < *P* ≤ 0.10.

## RESULTS

### Chemical Analysis

The analyzed total SBM AA concentration was similar across locations and the values were comparable to those presented in the NRC (2012). The corn AA profile was also similar across locations and, in general, slightly lower than NRC (2012) values. In general, DDGS used in Exp. 1 had the highest AA content and the DDGS used in Exp. 2, 3, and 4 had a similar AA profile. All DDGS sources had higher total AA content than the values reported in the NRC (2012), especially total Lys. The DDGS sources had variation in fiber and oil content; thus, the NE estimates were different for each source. The differences in ingredient composition across locations were accounted for in diet formulation and are not expected to have influenced the outcome of the study. The analyzed dietary crude protein, Ca, P, and neutral detergent fiber were consistent with formulated values (Tables 3–6).

There was variation in mycotoxin content in DDGS across locations (Table 2). The DDGS used in Exp. 1 had significant concentration of deoxynivalenol (DON) and total fumonisin, 1,047 and 6,347 ppb, respectively. Similarly, the DDGS used in Exp. 3 and 4 had high levels of DON (4,093 and 4,231 ppb, respectively) and contained detectible levels of zearalenone (328 and 274 ppb, respectively). The DDGS used in Exp. 2 did not contain particularly high levels of any mycotoxin.

### Experiment 1

There was a tendency (*P* = 0.086) for an SBM × DDGS interaction for G:F (Table 7). Gain-to-feed ratio increased and then decreased as SBM increased in diets without DDGS. However, in diets with DDGS, G:F was similar in pigs fed 32.5% and 37.5%, and both were better than those fed 27.5%. There was no evidence (*P* > 0.10) for interactions for ADG, ADFI, or CE. Pigs fed diets with DDGS had decreased (*P* < 0.01) ADG, ADFI, and final BW, as well as poorer CE (Table 8). Pigs fed increasing SBM had a tendency (*P* = 0.078) for a linear improvement in CE.

**Table 7.** Interactive effects of DDGS and SBM on growth performance of nursery pigs

	0% DDGS			25% DDGS				Probability, *P* <	
Item	27.5% SBM	32.5% SBM	37.5% SBM	27.5% SBM	32.5% SBM	37.5% SBM	SEM	DDGS × SBM linear	DDGS × SBM quadratic
Initial BW, kg									
Exp. 1^*a*^	10.5	10.6	10.5	10.6	10.6	10.5	0.20	0.758	0.926
Exp. 2^*b*^	11.7	11.7	11.7	11.7	11.6	11.7	0.18	0.999	0.736
Exp. 3^*c*^	12.5	12.5	12.5	12.5	12.5	12.5	0.25	0.845	0.875
Exp. 4^*d*^	12.3	12.3	12.3	12.3	12.3	12.3	0.57	0.992	0.984
Final BW, kg									
Exp. 1	25.4	25.8	25.0	23.3	24.3	23.6	0.54	0.251	0.595
Exp. 2	22.7	23.0	22.9	22.4	22.3	22.2	0.25	0.220	0.459
Exp. 3	26.2	26.1	25.9	25.5	24.8	24.5	0.38	0.013	0.271
Exp. 4	24.7	24.3	24.9	23.0	23.4	23.4	0.99	0.668	0.205
ADG, g									
Exp. 1	621	620	603	519	558	535	20.4	0.263	0.389
Exp. 2	524	539	533	510	508	497	6.9	0.063	0.568
Exp. 3	650	648	637	618	586	571	7.7	0.003	0.198
Exp. 4	592	570	598	510	531	529	21.5	0.553	0.076
ADFI, g									
Exp. 1	919	904	888	839	852	814	26.3	0.895	0.421
Exp. 2	794	798	783	767	771	732	11.3	0.190	0.476
Exp. 3	967	955	930	927	870	847	11.5	0.001	0.016
Exp. 4	869	835	858	786	802	782	32.9	0.813	0.111
G:F, g/kg									
Exp. 1	677	686	679	616	655	656	12.3	0.086	0.563
Exp. 2	660	676	681	665	661	679	5.1	0.460	0.039
Exp. 3	673	678	686	666	674	674	5.0	0.500	0.519
Exp. 4	682	685	698	651	664	675	11.1	0.675	0.690
CE, kcal/kg gain									
Exp. 1	3,829	3,780	3,819	4,231	3,967	3,980	80.9	0.102	0.454
Exp. 2	3,925	3,836	3,809	3,897	3,928	3,819	76.2	0.470	0.031
Exp. 3	3,852	3,821	3,778	3,895	3,846	3,846	28.8	0.615	0.477
Exp. 4	3,815	3,794	3,714	3,999	3,908	3,843	63.5	0.601	0.642

^*a*^A total of 296 pigs (initially 10.6 kg) were used in a 24-d study with four or five pigs per pen and 10 replicates per treatment.

^*b*^A total of 2,502 pigs (initially 11.7 kg) were used in a 21-d trial with 24–27 pigs per pen and 16 replicates per treatment.

^*c*^A total of 4,118 pigs (initially 12.5 kg) were used in a 21-d trial with 48–54 pigs per feeder (experimental unit) and 13 replicates per treatment.

^*d*^A total of 711 pigs (initially 12.3 kg) were used in a 21-d trial with 9 or 10 pigs per pen and 12 replicates per treatment.

**Table 8.** Main effects of DDGS and SBM on growth performance of nursery pigs

	DDGS				SBM				Probability, *P* <	
Item	0%	25%	SEM	Probability, *P* <	27.5%	32.5%	37.5%	SEM	Linear	Quadratic
Initial BW, kg										
Exp. 1^*a*^	10.5	10.6	0.184	0.602	10.5	10.6	10.5	0.19	0.727	0.514
Exp. 2^*b*^	11.7	11.7	0.162	0.980	11.7	11.7	11.7	0.17	0.951	0.763
Exp. 3^*c*^	12.5	12.5	0.244	0.779	12.5	12.5	12.5	0.25	0.462	0.559
Exp. 4^*d*^	12.3	12.3	0.561	0.947	12.3	12.3	12.3	0.57	0.988	0.991
Final BW, kg										
Exp. 1	25.4	23.7	0.461	0.001	24.3	25.0	24.3	0.48	0.838	0.014
Exp. 2	22.9	22.3	0.214	0.001	22.6	22.7	22.5	0.23	0.927	0.356
Exp. 3	26.1	25.0	0.359	0.001	25.9	25.5	25.2	0.36	0.001	0.459
Exp. 4	24.6	23.3	0.955	0.001	23.9	23.9	24.1	0.97	0.426	0.626
ADG, g										
Exp. 1	615	537	16.21	0.001	570	589	569	17.4	0.915	0.137
Exp. 2	532	505	5.02	0.001	517	524	515	5.6	0.726	0.127
Exp. 3	645	591	6.40	0.001	634	617	604	6.8	0.001	0.612
Exp. 4	587	523	19.32	0.001	551	550	564	19.9	0.271	0.500
ADFI, g										
Exp. 1	904	835	21.80	0.001	879	878	851	23.0	0.123	0.397
Exp. 2	792	757	8.41	0.001	780	784	757	9.2	0.015	0.057
Exp. 3	951	881	10.67	0.001	947	913	889	11.0	0.001	0.289
Exp. 4	854	790	30.03	0.001	827	819	820	30.8	0.666	0.727
G:F, g/kg										
Exp. 1	681	642	8.44	0.001	647	670	667	9.6	0.067	0.166
Exp. 2	672	668	3.51	0.290	663	668	680	4.0	0.001	0.456
Exp. 3	679	671	3.63	0.028	669	676	680	4.0	0.014	0.733
Exp. 4	688	663	8.24	0.001	666	675	687	9.0	0.027	0.841
CE, kcal/kg gain										
Exp. 1	3,809	4,059	55.33	0.001	4,030	3,873	3,899	63.0	0.078	0.152
Exp. 2	3,857	3,881	20.15	0.258	3,911	3,882	3,814	23.0	0.001	0.403
Exp. 3	3,817	3,863	20.96	0.025	3,874	3,834	3,812	23.1	0.013	0.657
Exp. 4	3,774	3,917	46.84	0.002	3,908	3,851	3,778	51.4	0.017	0.858

^*a*^A total of 296 pigs (initially 10.6 kg) were used in a 24-d study with four or five pigs per pen and 10 replicates per treatment.

^*b*^A total of 2,502 pigs (initially 11.7 kg) were used in a 21-d trial with 24–27 pigs per pen and 16 replicates per treatment.

^*c*^A total of 4,118 pigs (initially 12.5 kg) were used in a 21-d trial with 48–54 pigs per feeder (experimental unit) and 13 replicates per treatment.

^*d*^A total of 711 pigs (initially 12.3 kg) were used in a 21-d trial with 9 or 10 pigs per pen and 12 replicates per treatment.

### Experiment 2

There was an SBM × DDGS interaction (*P* = 0.039) for G:F (Table 7). Pigs fed diets without DDGS had increasing improvements in G:F as SBM concentration increased. However, for pigs fed diets with DDGS, increasing SBM from 27.5% to 32.5% resulted in similar G:F but it was improved for pigs fed diets with 37.5% SBM. A similar interaction (*P* = 0.032) was observed for CE. There was a tendency (*P* = 0.063) for an SBM × DDGS interaction for ADG, where ADG increased in pigs fed increasing SBM in diets without DDGS, whereas ADG decreased as SBM increased in diet with DDGS. There was no evidence (*P* > 0.10) for interactions for ADFI and final BW. Pigs fed diets with DDGS had decreased (*P* = 0.001) ADFI and final BW (Table 8). Increasing SBM resulted in a decrease (linear, *P* = 0.015) in ADFI.

### Experiment 3

There were SBM × DDGS interactions (*P* < 0.01) for ADG, ADFI, and final BW (Table 7). Pigs had decreased ADG, ADFI, and final BW as SBM increased; however, the magnitude of the decrease was greater for pigs fed diets with DDGS than those fed diets without DDGS. There was no evidence for interactions for G:F or CE. Pigs fed diets with DDGS had poorer (*P* ≤ 0.028) G:F and CE and those fed increasing SBM had improved (linear, *P* ≤ 0.014) G:F and CE (Table 8).

### Experiment 4

There was a tendency (*P* = 0.076) for an SBM × DDGS interaction for ADG (Table 7). Pigs fed diets without DDGS had decreased ADG when fed 32.5% SBM compared to 27.5% or 37.5% SBM, whereas pigs fed diets with DDGS had higher ADG when diets contained 27.5% or 37.5% SBM. There was no evidence (*P* > 0.10) for interactions for ADFI, G:F, or CE. Pigs fed diets containing DDGS had decreased (*P* ≤ 0.002) ADFI, G:F, and poorer CE (Table 8). Increasing SBM resulted in an improvement (linear, *P* ≤ 0.027) in G:F and CE.

### Culls and Mortality

In general, pigs used in all experiments were healthy and did not undergo major health challenges during the experimental period. The average cull rate was 0.7%, 0.5%, 0.2%, and 0% and the mortality rate was 0.7%, 0.3%, 0.4%, and 0% in Exp. 1–4, respectively (Table 9). Due to the low number of events, the statistical analysis for cull rate was not performed and only descriptive statistics are presented.

**Table 9.** Effects of DDGS and SBM on cull and mortality rate of nursery pigs^*a*,*b*^

	0% DDGS			25% DDGS		
Item	27.5% SBM	32.5% SBM	37.5% SBM	27.5% SBM	32.5% SBM	37.5% SBM
Culls, %						
Exp. 1	0.0	2.0	2.0	0.0	0.0	0.0
Exp. 2	0.5	0.7	0.7	0.0	0.5	0.5
Exp. 3	0.1	0.4	0.1	0.3	0.4	0.1
Exp. 4	0.0	0.0	0.0	0.0	0.0	0.0
Mortality, %						
Exp. 1	2.0	0.0	0.0	0.0	2.0	0.0
Exp. 2	0.2	0.0	0.2	0.0	0.2	1.0
Exp. 3	0.4	0.3	0.3	0.3	0.3	0.6
Exp. 4	0.0	0.0	0.0	0.0	0.0	0.0
Total, %						
Exp. 1	2.0	2.0	2.0	0.0	2.0	0.0
Exp. 2	0.7	0.7	1.0	0.0	0.7	1.5
Exp. 3	0.5	0.7	0.4	0.6	0.7	0.7
Exp. 4	0.0	0.0	0.0	0.0	0.0	0.0

^*a*^A total of 296, 2,502, 4,118, and 711 pigs were used in Exp. 1, 2, 3, and 4, respectively, in 21-d duration nursery trials.

^*b*^Descriptive data is presented. Due to the low number of events, statistical analysis was not performed.

## DISCUSSION

The United States is the world’s largest producer of soybeans, with an annual production of approximately 120 million tons in 2017, followed by Brazil and Argentina with 113 and 47 million tons, respectively (ASA, 2018). The majority of soybeans are destined to oil and SBM production, and almost 8 million tons of SBM were fed to pigs in the United States in 2017 (ASA, 2018). Typically, swine nutritionists formulate diets with dehulled solvent-extracted SBM, which contains approximately 47.5% crude protein and a balanced AA profile particularly rich in Lys, Thr, and Trp (NRC, 2012). These AA are limiting in typical swine diets and are relatively low in corn and wheat; thus, SBM complements their AA profiles well. Also, SBM AA digestibility is high for swine, with essential AA SID coefficients ranging from 85% to 94% (NRC, 2012). Finally, protein quality, expressed as AA concentration as a percentage of crude protein, is higher for SBM relative to other protein sources (Stein et al., 2013). Taken together, these characteristics contribute to the prevalent SBM use as a primary swine diet protein source in the United States and globally.

The addition of SBM is typically restricted to less than 20% of the diet in the period immediately postweaning due to a transient type II hypersensitivity reaction (Engle, 1994). This reaction is caused by antigenic proteins present in SBM, namely glycinin and conglycinin, and results in decreased growth performance (Li et al., 1990; Engle, 1994). Nevertheless, after initial exposure, there is little evidence for negative effect of feeding high SBM levels. Thus, it is not necessary to restrict its inclusion in late nursery diets or above approximately 11 kg BW.

Diets with high feed-grade AA inclusion are commonly used with the lower cost commercial availability of L-Lys, L-Thr, DL-Met or L-Met, L-Trp, and L-Val (Clark et al., 2017b; Menegat et al., 2019) at the expense of intact protein sources, such as SBM. Moreover, as our knowledge of the next limiting AA requirements, such as Ile (Clark et al., 2017a) and His (Cemin et al., 2018), develops and AA prices become more competitive, formulation strategies with higher inclusion of feed-grade AA are expected. Although the use of feed-grade AA has potential benefits regarding diet costs, research suggests that there may be benefits of feeding high levels of SBM, especially for health-challenged pigs. Porcine respiratory and reproductive syndrome is one of the most prevalent diseases of swine globally (Lunney et al., 2010) and causes estimated annual losses of over $600 million in the United States (Holtkamp et al., 2012). Therefore, strategies to mitigate the economic impact of PRRS can greatly benefit the swine industry. Johnston et al. (2010) were the first to describe the advantages of feeding high SBM for naturally PRRS-infected pigs. The authors observed a 10% improvement in ADG and 8% improvement in G:F for grow-finish pigs fed diets with 32% SBM compared to 21% SBM supplemented with feed-grade AA. Later, Rocha et al. (2013) observed that nursery pigs inoculated with PRRS virus had similar ADG but improved G:F when SBM was increased from 12.5% to 22.5% of the diet. This effect was observed during the first week postinoculation, but no differences were observed in subsequent periods. Conversely, Rochell et al. (2015) observed that PRRS-infected nursery pigs had improved growth when SBM increased from 17.5% to 29% of the diet, as well as lower serum PRRS virus load. In our study, only marginal improvements in ADG were observed with increasing SBM and a reduction was observed in some cases. It is important to note that the pigs used in the current experiments had relatively high health status, as evidenced by the low number of culls and mortality, and were not exposed to significant health challenges throughout the experimental period. Therefore, our results may not be directly comparable to previous research. Interestingly, Rochell et al. (2015) observed that pigs not infected with PRRS did not benefit from the high inclusion of SBM and even presented reduced ADG in some periods, which is in agreement with our findings. Taken together, it seems that pigs raised under high health conditions do not seem to benefit from high inclusions of SBM to the same extent as PRRS-infected pigs.

The reasons behind the benefits of feeding higher SBM diets to pigs are unclear. The improvement in growth performance of PRRS-infected pigs fed increasing SBM does not seem to be related to changes in nutrient or AA digestibility (Schweer et al., 2018). One of the modes of action could be explained by the presence of bioactive components in SBM, namely isoflavones and saponins. A review of these components was recently published (Smith and Dilger, 2018) and will not be described in detail. Briefly, isoflavones and saponins have been reported to have anti-inflammatory, antioxidant, and antiviral properties, as well as the ability to modulate intestinal permeability. However, the available research shows uncertainty regarding the effects of isoflavones. In a wean-to-finish trial, Kuhn et al. (2004) compared SBM and soy protein concentrate, an ingredient with markedly lower isoflavones relative to SBM. The authors observed no evidence for differences in growth performance in any stage of production, although plasma isoflavone concentration was higher in pigs fed SBM than those fed soy protein concentrate. On the other hand, in a grow-finish study, Payne et al. (2001) observed reduced growth in late finishing pigs fed soy protein concentrate diets supplemented with isoflavones compared to pigs fed soy protein concentrate- or SBM-based diets but no significant differences overall. It appears that isoflavones could be more beneficial when fed to health-challenged pigs, but results are also inconsistent. Greiner et al. (2001a, b) observed improvements in performance of PRRS-positive pigs driven by increasing isoflavones but mostly during periods of peak viremia. Conversely, Smith et al. (2017) evaluated diets with or without supplementation of isoflavones for PRRS-infected nursery pigs and found no improvements in growth performance, although some immunological changes were observed.

A consistent finding in our experiments was an improvement in G:F and CE as SBM increased. Yet again, the reasons for these responses are unclear as they could be driven by the intrinsic bioactive components but also by an underestimation of the energy value assigned for SBM (Boyd et al., 2011; Li et al., 2017). Underestimating or overestimating NE can be detected if pigs fed diets with increasing amount of a test ingredient present differences in G:F or CE (De Jong et al., 2014; Gonçalves et al., 2016). Our findings suggest that the energy value assigned for SBM could have been underestimated. The NRC (2012) NE estimate for SBM is 2,087 kcal/kg or 78% of corn NE. Our diets were assuming SBM had 2,351 kcal/kg or 88% of corn NE and balanced for NE. Therefore, this suggests that the NRC (2012) considerably underestimates the NE value of SBM, and this has important ramifications in diet formulation as it increases the value of SBM. A comparable result was reported by Cemin et al. (2019), who also formulated diets with SBM NE at 88% of corn NE and observed approximately 4% improvement in G:F of nursery pigs when SBM inclusion increased from 27% to 35%, suggesting an SBM NE value greater than corn. Moran et al. (2017) conducted two trials evaluating increasing SBM for nursery pigs. In the first trial, pigs were PRRS negative and the authors observed a consistent improvement in G:F in agreement with our findings. However, the results were not repeated in a subsequent study with pigs originated from a PRRS-positive sow farm, where increasing SBM in the diet did not improve growth performance but reduced the percentage of pigs removed for medical treatment from 11.1% to 8.4%.

It is unclear why growth performance was negatively impacted with high amounts of SBM in some of the current experiments, especially when diets contained DDGS. The available research generally does not agree with this finding; as most of the studies found no change or improvements in ADG with increasing SBM, it is important to note that the current study evaluated higher SBM additions than the majority of previous research. Therefore, a possible explanation for our finding is the dietary crude protein level. The diets with the highest inclusion of SBM contained on average 27% crude protein. It is well known that pigs do not have a crude protein requirement but rather a need for AA. Protein or AA provided in excess will be deaminated and excreted, thus representing an inefficient use of nutrients and an energy cost to the animal (Van Milgen and Dourmad, 2015). Moreover, undigested protein can contribute to the proliferation of nitrogen-utilizing pathogenic bacteria in the gastrointestinal tract (Ball and Aherne, 1987), and high crude protein diets have been shown to increase the incidence of diarrhea in nursery pigs (Heo et al., 2009). Finally, dietary crude protein has the ability to impact gut morphology and gut microbiota (Opapeju et al., 2009). Therefore, it may be important to limit the inclusion of SBM, especially in diets formulated with DDGS, to avoid excess dietary crude protein. Taken together, it is challenging to identify the reason for the decreased growth of pigs fed high-protein diets, and it is likely driven by multiple factors.

Our experiments showed that pigs fed diets with 25% DDGS had decreased growth performance compared to those fed corn-SBM diets. In contrast, the literature suggests that feeding DDGS to late nursery pigs is typically not detrimental to growth performance (Stein and Shurson, 2009). Whitney and Shurson (2004) observed no evidence for differences in late nursery performance for pigs fed up to 25% DDGS. A similar observation was made by Jones et al. (2010) when feeding up to 30% DDGS and Cemin et al. (2019) when diets contained 23% DDGS. The negative response to DDGS found in the current study could have been driven by the higher fiber content of the ingredient, although the DDGS sources used in our experiments were comparable in fiber content to previous research (Whitney and Shurson, 2004; Jones et al., 2010; Cemin et al., 2019). It could also be hypothesized that the energy value of DDGS was underestimated, which would help explain the G:F and CE responses observed in three of the four experiments. The presence of mycotoxins could also explain the reduced growth performance observed in pigs fed diets with 25% DDGS. The U.S. Food and Drug Administration (FDA) recommends that feed ingredients contain less than 5,000 ppb DON and that these ingredients do not exceed 20% of the diet for a maximum of 1,000 ppb DON in complete feed (FDA, 2010). The DDGS used in the current experiments contained 1,047, 825, 4,093, and 4,231 ppb DON in Exp. 1, 2, 3, and 4, respectively. These levels are below the FDA recommendation but DDGS was included at 25% of the diet, thus resulting in dietary concentrations slightly greater than 1,000 ppb in Exp. 3 and 4. Furthermore, the recommended total fumonisin level in feed ingredients is 20,000 ppb and these ingredients do not exceed 50% of the diet (FDA, 2001). Therefore, all DDGS sources were under the recommended levels, with the highest concentration of total fumonisin observed in the DDGS used in Exp. 1 (6,347 ppb). Although the individual mycotoxin levels were generally below the recommended levels by the FDA, some mycotoxins can interact and potentially present additive or synergistic toxicity (Huff et al., 1988; Pierron et al., 2016); thus, their impact on growth performance cannot be predicted upon individual concentrations. Other factors for the negative DDGS response include variability among sources (Spiehs et al., 2002), changes in palatability (Hastad et al., 2005), or feed intake limitation to the lower bulk density (Ndou et al., 2012).

In conclusion, a common observation from these studies is that DDGS generally reduced growth performance, possibly influenced by mycotoxin levels. On the other hand, increasing addition of SBM from 27.5% to 37.5% of the diet did not result in major changes in ADG but consistently improved G:F and CE. The underlying mechanism for this response is unclear but could be driven by intrinsic SBM components, such as isoflavones, or by underestimating SBM energy value.
